# The Louisville Medical News

**Published:** 1876-01

**Authors:** 


The Louisville Medical News, a weekly journal of medicine and surgery, volume 1, No. 1, we find upon our table. This new candidate for the patronage of the profession is edited by Prof. Richard O. Cowling, of the University of Louisville, and Dr. William H. Galt, and published by John P. Morton & Co., at the low price of $3.10 per annum in advance, postage paid. We have read the initial number with interest, and gladly welcome it to our exchange list. The work of the publishers is very handsomely done, and we trust that the *News* will meet with the favor from the profession which it certainly deserves. We know of no weekly in the country which offers so much, in so good style, at so low price.

